# Neuroprotective potential of selected lichen compounds on mouse neuroblastoma (N2a) cells

**DOI:** 10.17179/excli2020-3130

**Published:** 2021-02-24

**Authors:** Sachin Mapari, Subhash Gaikwad, Roshni Khare, Muntjeeb Syed, Pooja Doshi, Bhaskar C. Behera

**Affiliations:** 1 Agharkar Research Institute, G.G. Agarkar Road, Pune, India; 2 Department of Chemistry, Savitribai Phule Pune University, Pune, India

## ⁯

***Dear Editor, ***

Alzheimer's disease (AD), a prevalent form of dementia, affects a significant number of people in the world. According to some reports, the treatment of AD with synthetic AChE inhibitors has adverse effects like hepatotoxicity and gastrointestinal problems (Roseiro et al. 2012[5]). Only a handful of studies regarding intracellular ROS modulation by lichen extracts and metabolites have been performed (Fernández-Moriano et al., 2016[3]). Our experiment was directed to ascertain lichen and its metabolites with potential antioxidant activities and its possible neuroprotective potential under H_2_O_2_ induced oxidative stress in mouse neuroblastoma cells (N2a).

Lichen species *Flavoparmelia caperata*, *Flavopunctelia flaventior *and *Heterodermia leucomelos *were collected from Western Himalaya, India. Major compounds such as Protocetraric acid, Lecanoric acid and Zeorin were isolated by PTLC and confirmed with HPLC analysis (Behera et al., 2012[1]; Verma et al., 2012[7]). The antioxidative potential of metabolites was evaluated by scavenging of DPPH, ABTS, H_2_O_2,_ and FRAP. The IC_50 _value of all metabolites was found to be higher than the standard antioxidants Ascorbic acid and Trolox. AChE and BChE inhibition was determined using Ellman's method with slight modifications (Ingkaninan et al., 2000[4]). The results indicated that a higher concentration of metabolites achieved 50 % inhibition of AChE and BChE as compared to the standard inhibitor Galanthamine (Table 1(Tab. 1)).

**Figure 1:**
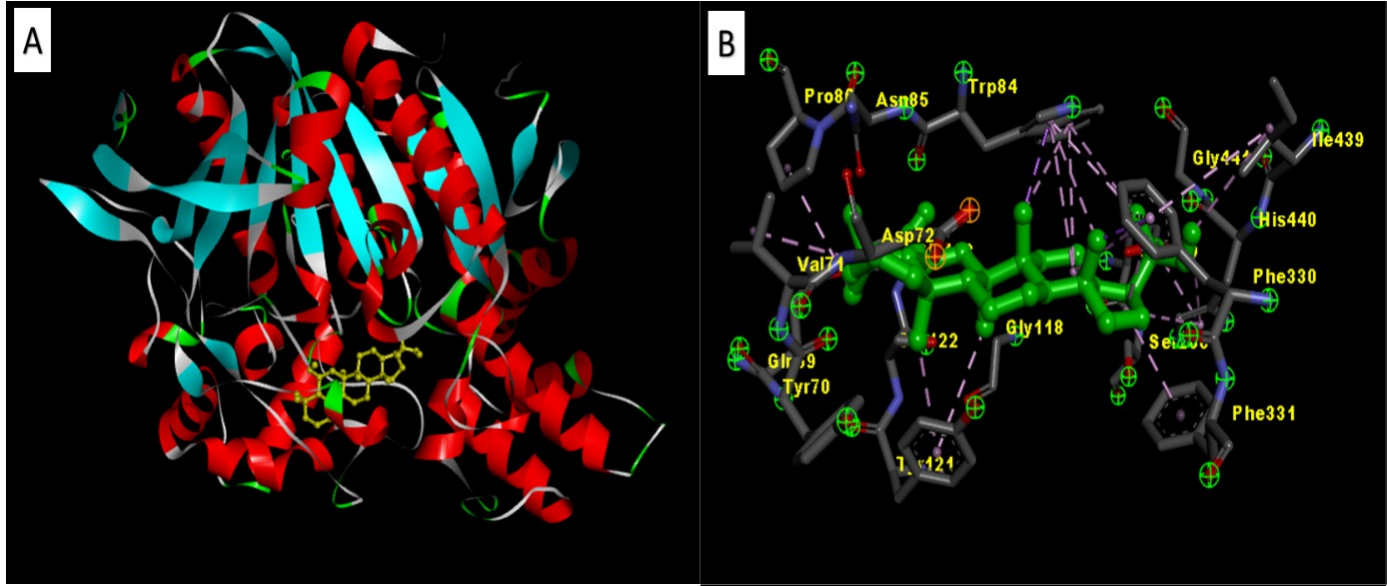
[A] 3D structure of the docked Zeorin against AChE, [B] Zeorin specific interaction with AChE

The AChE-Zeorin docking study was conducted using AutoDock 1.5.6, Discovery Studio visualizer 2.1 and Cygwin64. From the dock score analysis, Zeorin showed the highest dock score of 32.14 kcal/mol indicating significant AChE-Zeorin interactions (Figure 1(Fig. 1)). Zeorin molecules' specific interaction with AChE leads to its conformational change. The high dock score is a sign of immense binding efficiency between the enzyme and ligand (Sheeja Malar et al., 2017[6]).

**Figure 2:**
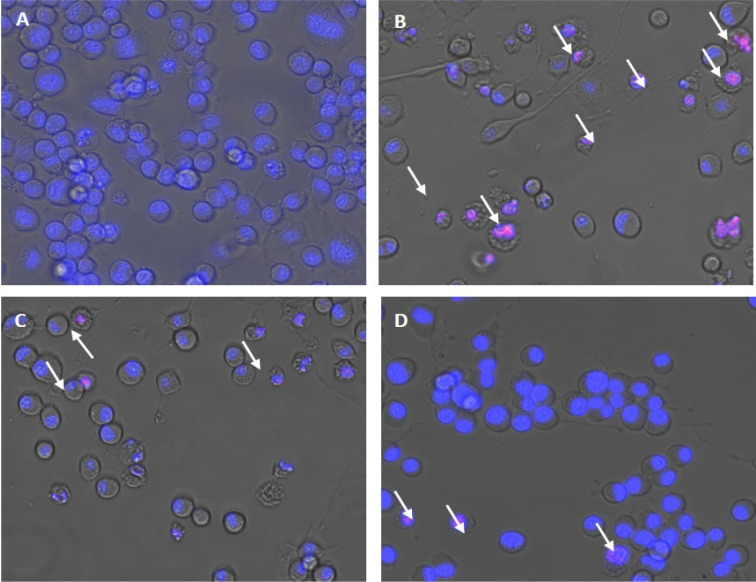
H_2_O_2_-induced apoptosis in Neuro-2a cells. (A) Control, (B) the number of cells decreased after H_2_O_2_ (150 µM) treatment, (C) H_2_O_2_ (150 µM) treated + Lecanoric acid (12.5 µg/ml) and (D) H_2_O_2_ (150 µM) treated + Zeorin (6 µg/ml). Images are representative of three independent experiments.

Mouse neuroblastoma cell line (N2a) was obtained from National Centre for Cell Sciences (NCCS) cell repository, Pune, India. It was observed that treatment with H_2_O_2 _(150 µM) reduced the cell viability to 60 %. Besides, pre-treatment of the non-cytotoxic doses obtained from the cell viability assay significantly reduced the effect of H_2_O_2 _induced toxicity and enhanced the viability of neuron cells. Lecanoric acid at a low concentration of 1 μg/ml and Zeorin at 6 μg/ml increased the cell viability up to 20 %. This increase in cell growth may be on account of a reduction in oxidative radical generation. Our results were consistent with the ones reporting the ameliorative effect of Evernic acid on intracellular ROS by their cytoprotective property via oxidative stress reduction (Fernández-Moriano et al., 2017[2]). The changes in cellular morphology after treatment were observed using Floid cell imaging (20X). The staining of Neuro-2a cells was done by ReadyProbes Cell Viability Imaging Kit, with blue indicating live cells and red indicating cell death (Figure 2(Fig. 2)). 

The results of our *in silico* work were justified by *in vitro* studies, which showed potent dual cholinesterase inhibition. The lichen compound Zeorin showed promising neuroprotective potential via antioxidation, AChE inhibition and cytoprotective activity against H_2_O_2-_induced toxicity in mouse Neuroblastoma (N2a) cell line. Our results indicate that Zeorin, being a natural resource is a better choice of treatment for AD as it exhibits significant neuroprotection. However, exhaustive studies are needed to provide sufficient insights on the mechanism/pathway of lichen compound Zeorin in neuroprotection ability.

## Acknowledgements

This work was supported by the Agharkar Research Institute, Pune, SERB, Govt. of India, New Delhi (File No. YSS/2014/000883) and DIC, MHRD, Govt. of Maharashtra. 

## Conflict of interest

All authors declare no conflict of interest. 

## Figures and Tables

**Table 1:**
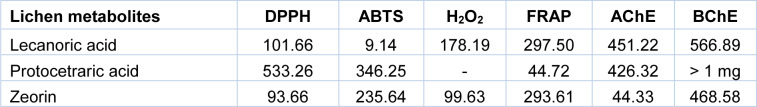
IC_50_ (μg/mL) value of lichen metabolites showing 50 % scavenging in antioxidant and cholinesterase inhibition. Data presented are the mean of three consecutive readings of samples in the assays performed. The mean value of each concentration of different lichen metabolite was used for IC_50_ value calculation by linear regression between metabolite concentrations versus biological activities.
